# Vessels characteristics in familial exudative vitreoretinopathy and retinopathy of prematurity based on deep convolutional neural networks

**DOI:** 10.3389/fped.2023.1252875

**Published:** 2023-08-24

**Authors:** Xinyi Deng, Kun Chen, Yijing Chen, Ziyi Xiang, Shian Zhang, Lijun Shen, Mingzhai Sun, Lingzhi Cai

**Affiliations:** ^1^ Center for Rehabilitation Medicine, Department of Ophthalmology, Zhejiang Provincial People’s Hospital (Affiliated People’s Hospital, Hangzhou Medical College), Hangzhou, China; ^2^ Department of Precision Machinery and Instrumentation, University of Science and Technology of China, Hefei, China; ^3^ Department of Retina Center, Eye Hospital of Wenzhou Medical University, Wenzhou, China; ^4^ Affiliated Hangzhou First People’s Hospital, Zhejiang University School of Medicine, Hangzhou, China

**Keywords:** deep learning, retinopathy of prematurity, familial exudative vitreoretinopathy, vascular morphology, retina

## Abstract

**Purpose:**

The purpose of this study was to investigate the quantitative retinal vascular morphological characteristics of Retinopathy of Prematurity (ROP) and Familial Exudative Vitreoretinopathy (FEVR) in the newborn by the application of a deep learning network with artificial intelligence.

**Methods:**

Standard 130-degree fundus photographs centered on the optic disc were taken in the newborns. The deep learning network provided segmentation of the retinal vessels and the optic disc (OD). Based on the vessel segmentation, the vascular morphological characteristics, including avascular area, vessel angle, vessel density, fractal dimension (FD), and tortuosity, were automatically evaluated.

**Results:**

201 eyes of FEVR, 289 eyes of ROP, and 195 eyes of healthy individuals were included in this study. The deep learning system of blood vessel segmentation had a sensitivity of 72% and a specificity of 99%. The vessel angle in the FEVR group was significantly smaller than that in the normal group and ROP group (37.43 ± 5.43 vs. 39.40 ± 5.61, 39.50 ± 5.58, *P* = 0.001, < 0.001 respectively). The normal group had the lowest vessel density, the ROP group was in between, and the FEVR group had the highest (2.64 ± 0.85, 2.97 ± 0.92, 3.37 ± 0.88 respectively). The FD was smaller in controls than in the FEVR and ROP groups (0.984 ± 0.039, 1.018 ± 0.039 and 1.016 ± 0.044 respectively, *P* < 0.001). The ROP group had the most tortuous vessels, while the FEVR group had the stiffest vessels, the controls were in the middle (11.61 ± 3.17, 8.37 ± 2.33 and 7.72 ± 1.57 respectively, *P* < 0.001).

**Conclusions:**

The deep learning technology used in this study has good performance in the quantitative analysis of vascular morphological characteristics in fundus photography. Vascular morphology was different in the newborns of FEVR and ROP compared to healthy individuals, which showed great clinical value for the differential diagnosis of ROP and FEVR.

## 1. Introduction

Congenital eye diseases are the leading cause of vision loss and blindness in infants and young children, which could cause heavy social and economic burdens worldwide (1). Congenital retinal diseases in infants include retinopathy of prematurity (ROP), familial exudative retinopathy (FEVR), retinoblastoma, and other developmental abnormalities.

With the increasing survival rates of very preterm and very low birth weight infants, the prevalence and severity of ROP are also increasing. FEVR is an inherited retinal vascular development disease, which presented resembling ROP (2). Unlike other dysplastic diseases, infantile retinal diseases are difficult to recognize with the naked eye and often progress to an advanced stage before being detected. Since the first 4–6 months period is crucial for the development of neonatal eyes, most of the affected infants would miss the best time for treatment. Therefore, early screening and diagnosis play the key role to preventing vision loss and blindness in infants and young children. The American Academy of Pediatrics (AAP) recommended screening for all infants born before 30 weeks gestational age or with a birth weight less than 1,500 g, as well as infants with additional risk factors (3). In addition, other than screening for prematurity, screening for full-term infants is equally important.

However, the temporal periphery vascular pattern in ROP and FEVR is remarkably similar (4). In clinical practice, the differential diagnosis is commonly based on medical history and fluorescein angiography (FA) (4, 5). In the past decade, FA in infants has been increasingly utilized to visualize the infant's retina without the need for anesthesia and intravenous injections. Through FA, ROP and FEVR lesions that are difficult to distinguish on color fundus photography and indirect ophthalmoscopy could be differentiated, as the former showing discrete and homogeneous vascular-avascular junctions, while the latter typically shows bulbous vascular terminals, abnormal branching patterns, and venous-venous shunting (6). Although FA for infants has been proven to be safe and effective, it remains challenging in terms of the shortage of pediatric ophthalmologists and the difficulties of nursing infants (7).

In recent years, the development of artificial intelligence has grown rapidly with the development of computer science as well as high-performance graphics processors. Artificial intelligence (AI) is a branch of computer science that refers to the use of computers to simulate human intelligence with only minimal human intervention (8). To date, most studies have focused on computer-based systems for the identification and diagnosis of plus disease in ROP. In 2016, Worrall et al. (9) first used Convolutional Neural Networks (CNNs) that can automatically analyze image features for automatic plus disease diagnosis. Our study applied CNN and deep learning to fundus images of preterm infants for automatic plus disease diagnosis and achieved a sensitivity of 95.1% and a specificity of 97.8% (10). Ye et al. (11) proposed an automated diagnostic system for FEVR that combines deep learning and available clinical evidence. The system focused on the evaluation of retinal vascular features in Ultra-wide-field fundus images to diagnose FEVR with quantitative measurement, enabling accurate and non-invasive diagnosis, including early stages of FEVR.

Therefore, the purpose of this study was to quantitatively analyze the vascular morphological characteristics of infants with ROP and FEVR using an artificial intelligence approach, hoping to provide insights into the differential diagnosis and early intervention of these diseases.

## 2. Material and methods

### 2.1. Participants

This study adhered to the Declaration of Helsinki and received approval by the ethics review board of Zhejiang Provincial People's Hospital. All the infants presented to the ophthalmology clinic from March 2022 to January 2023 were reviewed, and infants with a clinical diagnosis of ROP (289 eyes) or FEVR (201 eyes) were included in this study. Healthy full-term infants (195 eyes) were also included as a control group.

Exclusion criteria were vitreous hemorrhage, history of retinal laser photocoagulation or vitreous injection, or any opacity of the refractive media that may affect the image quality.

### 2.2. Fundus imaging

All the infants underwent standard contact wide-angle fundus imaging after pupil dilatation. The collected image size was 1,600⊆1,200 pixels with a 130-degree fundus angle. Posterior fundus images centered on the optic disc were collected to avoid bias caused by different capture perspectives.

### 2.3. Artificial intelligence analysis of vascular characteristic

**Figure 1:**
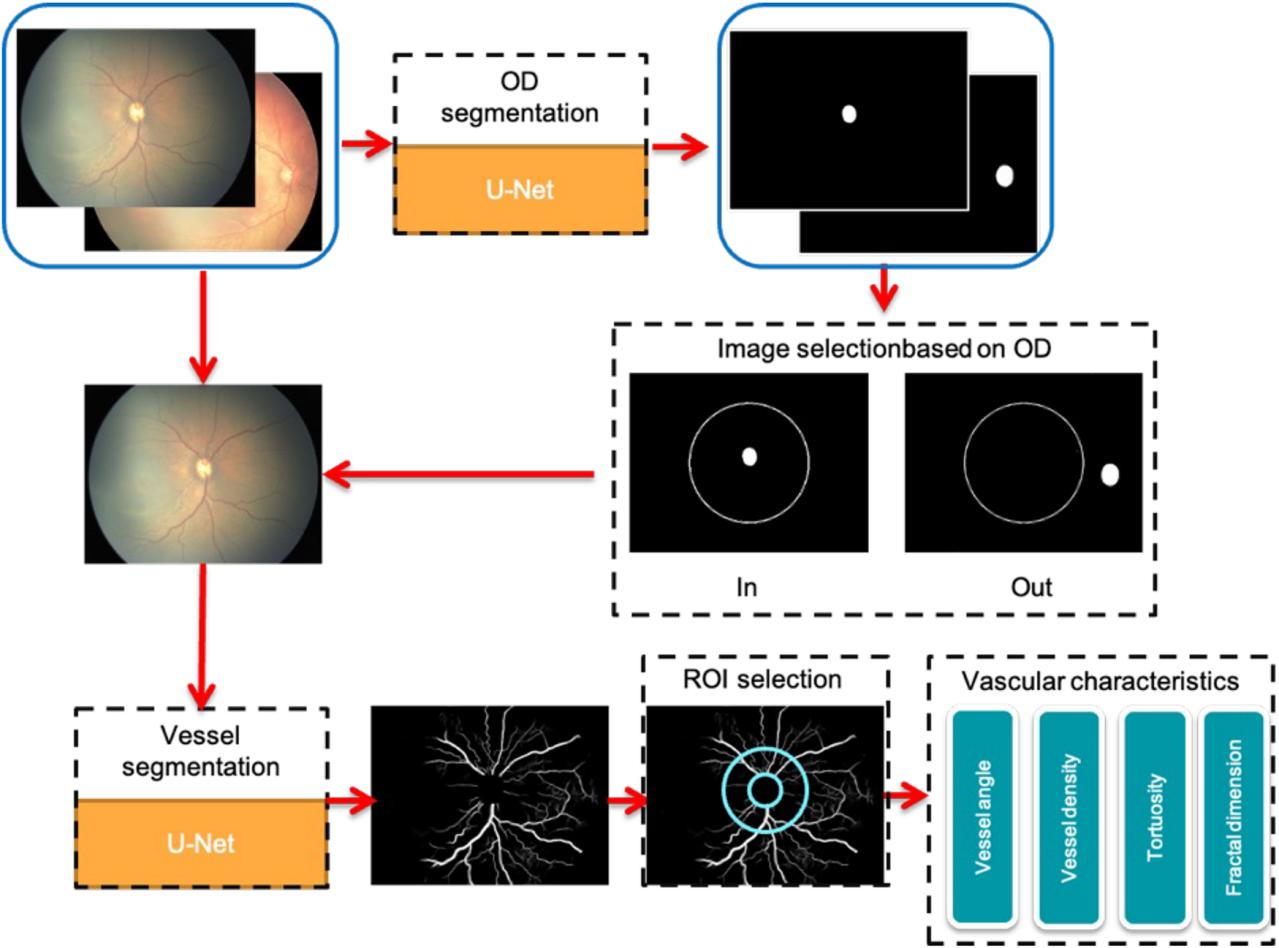
Overview of the data flow structure. OD, optic disc; ROI, region of interest.

The flowchart shown in Figure 1 illustrates the data processing steps used in our study. The specific method of constructing the optic disc and vascular segmentation network is detailed in our previous study (10). First, the retinal images were subjected to optic disc segmentation using a U-Net based network (12). The optic disc center was then calculated based on the segmentation result, and the minimum enclosing circle of the optic disc was fitted. A circle is then drawn with the image center as the center and one-quarter of the image width as the radius, and the optic disc center was checked whether it falls within this circle. The images with the optic disc center located within this circle were selected for blood vessel segmentation based on a U-Net. The concentric ring region of interest (ROI) was defined with the optic disc center as the center and 2–4 times the minimum enclosing circle radius of the optic disc as the radius, in which the vascular analysis is performed, including measurements of blood vessel angle, density, tortuosity, and fractal dimension.

The vessel angle refers to the angle formed between two blood vessels that arise from a bifurcation. We used the Baker et al. (13) definition to evaluate the vessel angle, specifically computing the vessel angle at the branching points. Retinal blood vessel density was calculated as the ratio of vessel area to the area of the fundus image. The fractal dimension serves as a comprehensive measure of the intricate branching pattern exhibited by the retinal vascular tree (14). Higher values of the fractal dimension indicate a more intricate branching pattern. To evaluate the fractal dimension of the fundus vessels, we utilized the box-counting method proposed by Mainster & Martin (15) and Stosic & Stosic (16). This method allowed us to quantify the fractal dimension by analyzing the distribution of vessel segments within the retinal images. Tortuosity is a parameter employed to assess the extent of curvature exhibited by blood vessels. Greater tortuosity values indicate a higher degree of curvature in the blood vessels. Various definitions of tortuosity have been proposed (17, 18). In our study, we adopted the measurement of tortuosity known as arc length-normalized total squared curvature, initially introduced by Hart et al. (17), which was subsequently refined by Turior et al. (19). This approach allows for a more accurate evaluation of tortuosity by considering the normalized curvature of the vessel segments along their arc length.

### 2.4. Statistical analysis

Statistical analysis was performed using SPSS software (version 26.0, SPSS Inc, Chicago, IL, USA). All data were presented in the form of means ± standard deviations. The vascular morphological characteristics were compared between the groups using the *χ*^2^ test or one-way analysis of variance. *P*-values <0.05 were considered statistically significant.

## 3. Results

### 3.1. Retinal vascular segmentation performance

**Figure 2:**
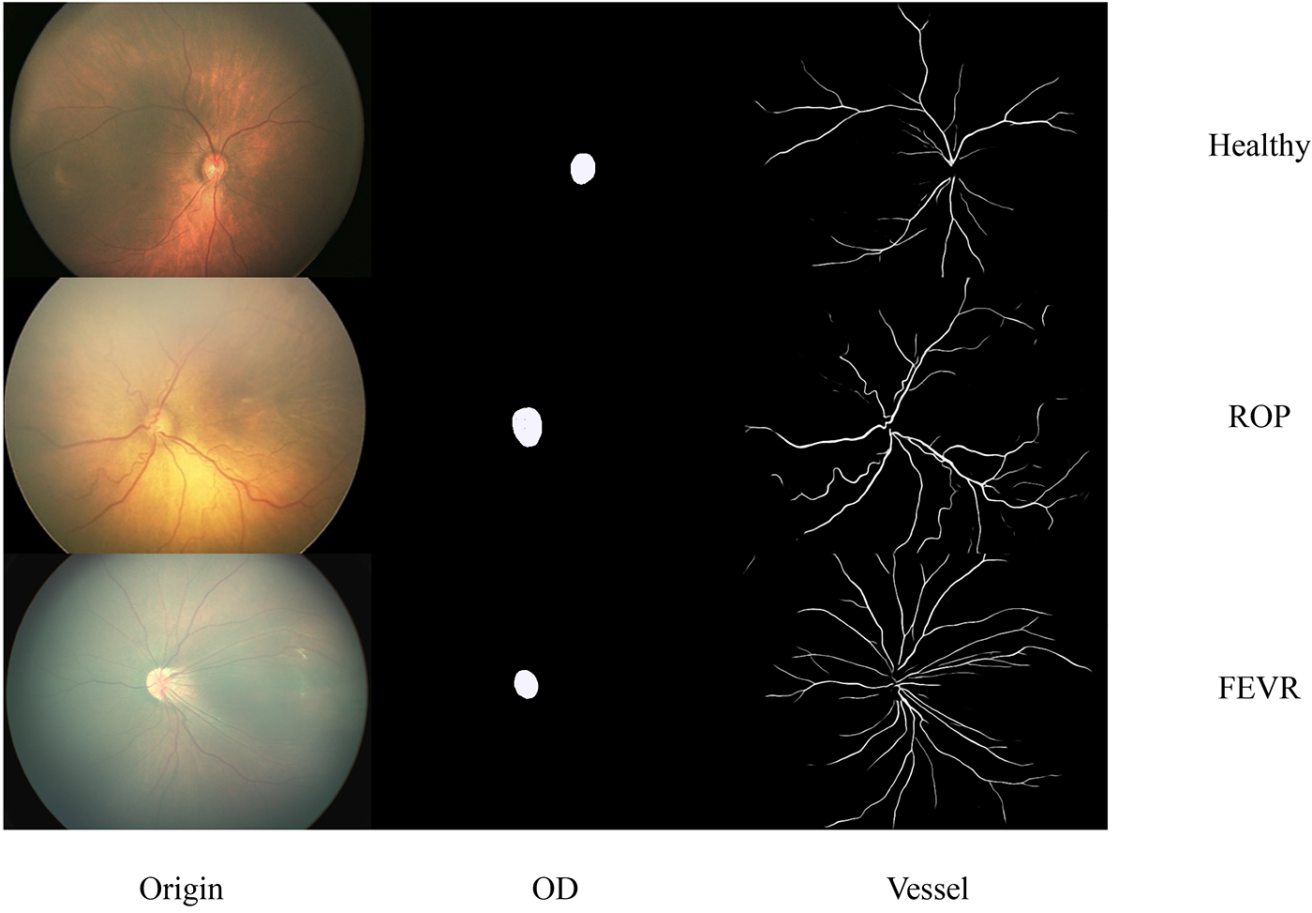
Representative images of original, optic disc segmented and retinal blood vessels for the healthy, ROP and FEVR eyes. OD, optic disc; ROP, retinopathy of prematurity; FEVR, familial exudative vitreoretinopathy.

The segmentation results of the optic disc and retinal blood vessels for the three groups are shown in Figure 2. The sensitivity and specificity for vascular segmentation were 0.72 and 0.99, respectively. For optic disc segmentation, the sensitivity was 0.94 and the specificity was 0.99.

### 3.2. Vessel angle

The difference in vessel angle was statistically significant between the three groups (*P* < 0.001). The vessel angle in the FEVR group was significantly smaller than that in the normal group and ROP group (37.43 ± 5.43 vs. 39.40 ± 5.61, 39.50 ± 5.58, *P* = 0.001, *P*<0.001 respectively). The vessel angle had no significant differences between ROP and the normal group (*P* = 0.980).

### 3.3. Vessel density

The difference in vessel density among the three groups was statistically significant, *P* < 0.001. The normal group had the lowest vessel density, the ROP group was in between, and the FEVR group had the highest (2.64 ± 0.85, 2.97 ± 0.92, 3.37 ± 0.88 respectively). The differences between the groups were all significantly different (all *P* < 0.001).

### 3.4. Fractal dimension

Fractal dimension was statistically different among the three groups, *P* < 0.001. The fractal dimension of the normal group was smaller than that of the ROP and FEVR groups (0.984 ± 0.039, 1.018 ± 0.039, 1.016 ± 0.044 respectively, both *P* < 0.001).

### 3.5. Vascular tortuosity

**Figure 3:**
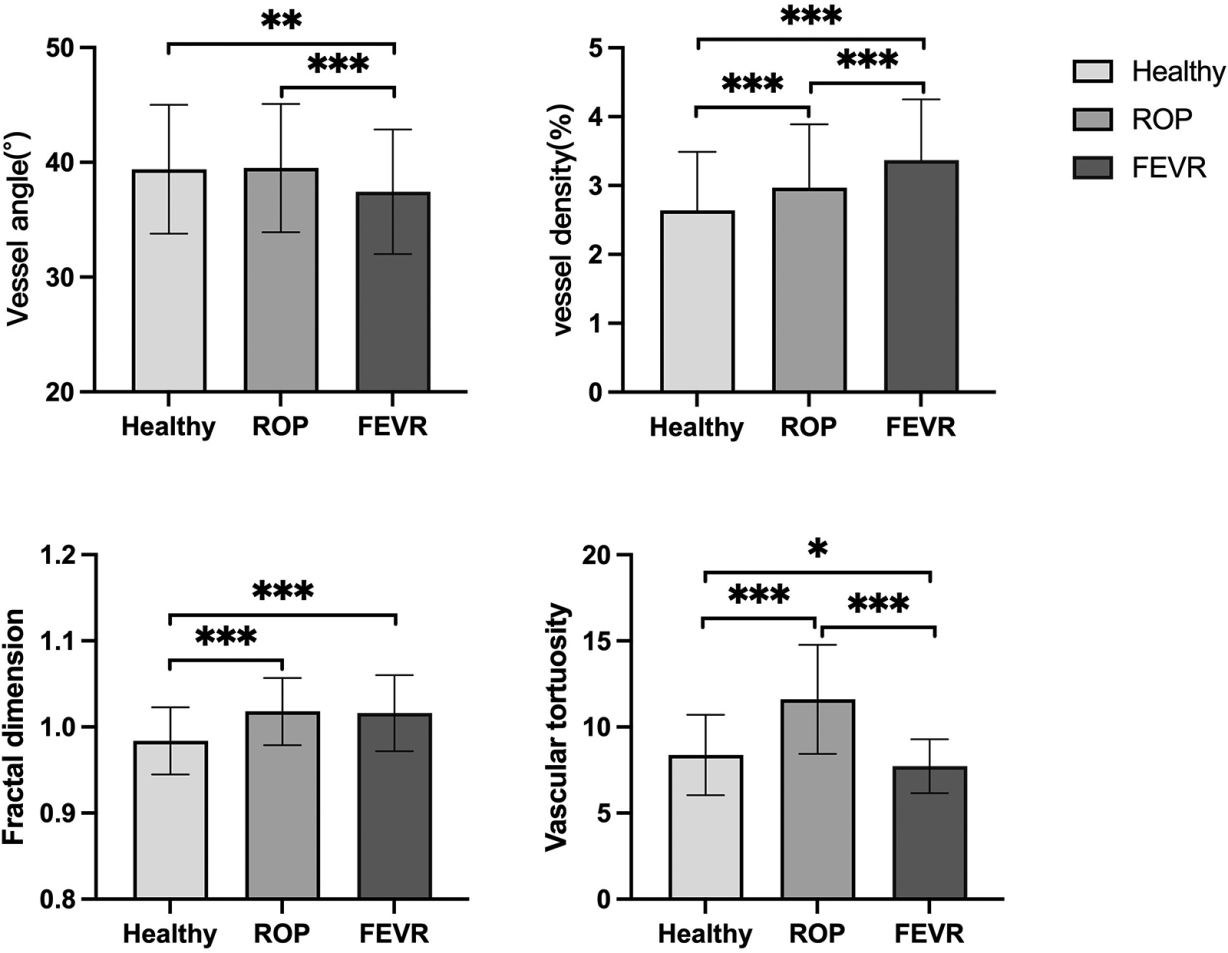
Differences in morphological characteristics among healthy, ROP and FEVR groups. ROP, retinopathy of prematurity; FEVR, familial exudative vitreoretinopathy. *P < 0.05, **P < 0.01, ***P < 0.001.

Among the three groups, the largest tortuosity appeared in the ROP group, while the FEVR group had the smallest value of tortuosity (11.61 ± 3.17, 8.37 ± 2.33, 7.72 ± 1.57 respectively, *P* < 0.001). Vascular morphologic parameters and intragroup comparisons among the three groups were shown in Table 1 and Figure 3, respectively.

**Table 1:** Clinical morphological characteristics in ROP, FEVR and healthy groups.

Morphological Characteristics	ROP (*n* = 289)	FEVR (*n* = 201)	Healthy group (*n* = 195)	*P*
Vessel angle (°)	39.50 ± 5.58	37.43 ± 5.43	39.40 ± 5.61	<0.001
Vessel density (%)	2.97 ± 0.92	3.37 ± 0.88	2.64 ± 0.85	<0.001
Fractal dimension	1.018 ± 0.039	1.016 ± 0.044	0.984 ± 0.039	<0.001
Vascular tortuosity (*10^−3^)	11.61 ± 3.17	7.72 ± 1.57	8.37 ± 2.33	<0.001

*n*, number of eyes; ROP, retinopathy of prematurity; FEVR, familial exudative vitreoretinopathy.

## 4. Discussion

In the present study, we used a deep learning system to automatically quantify and analyze the posterior retinal vascular morphology in the eyes of ROP, FEVR, and full-term newborns. We demonstrated that vessel morphological parameters (including vessel angle, vessel density, fractal dimension, and vessel tortuosity) were significantly different between the three groups of infants. We showed that ROP and FEVR are significantly different in vascular morphology and could be identified by artificial intelligence systems.

In the current study, a reduced vascular angle was found in the eyes of patients with FEVR compared to those with ROP and healthy full-term infants. We suggested that the decrease in vessel angle was possibly derived from the increased traction on the vitreoretinal interface. Lee et al. (20) analyzed the images on handheld OCT of young children with FEVR and revealed prominent retinal displacement and traction. Contraction of the strongly adherent vitreous further provoked the characteristic macular ectopia and retinal traction complications, especially seen in young patients with FEVR. In addition, Wang et al. (21) suggested that Ultra-Wide-Field OCTA could contribute to the detection of abnormalities at the temporal mid-peripheral vitreoretinal interface of patients with early stages of FEVR, which resulted in superficial retinal vascular straightening and a reduction in vessel angle. In agreement with our study results, Shao et al. (22) demonstrated a reduced angle of retinal arteries in FEVR patients in ultra-wide-field fundus imaging.

In the previous study by our team (10), it was demonstrated that due to the dilated and tortuous nature of the ROP retinal vessels, the vascular density increased with increasing severity of the plus disease. However, studies using OCTA revealed a reduction in both superficial and deep macular capillary vessel density in the eyes of patients with FEVR (23, 24). We speculated that this difference stems from the difference in measurement method and the age of the subjects.

The fractal dimension numerically measures the subtle distortions of tissue structure in pathological states and has become one of the new biomarkers in the field of neuroscience (25). In the field of ophthalmology, the fractal dimension has been used to detect early diabetic retinopathy and other retinal diseases (26). In addition, the fractal dimension has been proven to increase with the severity of diabetic retinopathy (27). Our study used the fractal dimension to quantify the complexity of retinal blood vessels in fundus imaging in infants. The results indicated that retinal vascular complexity was elevated in both FEVR and ROP infants compared to healthy full-term infants.

Traction on the temporal side of the retina in infants with FEVR causes straightening of the vessels. Hsu et al. observed straightening of the macular vasculature in OCTA imaging of patients with FEVR (24). Chen et al. (28) investigated fundus imaging in patients with asymptomatic mild FEVR and found increased vascular branching and straightening of peripheral vascular branches in 98.4% of eyes, which was also consistent with the results of our previous study (11). Vascular endothelial growth factor (VEGF) is the primary mediator of pathological angiogenesis in ROP (29). A study by Liang et al. found that elevated aqueous VEGF levels were positively correlated with ROP severity and venous tortuosity in zone Ⅰ (29). It has also been proposed that the mean and highest vascular tortuosity of fundus images of infants would be used to predict the need for treatment of ROP with a high level of sensitivity and specificity (30). Furthermore, Chen et al. suggested that vascular tortuosity following intravitreal injection of anti-VEGF for ROP is indicative of potential ROP lesion reactivation (31). Therefore, we suggested that the opposite trend of retinal vascular tortuosity in infants with FEVR and ROP indicates a different pathological process between these two diseases.

This study has several limitations. First, given the retrospective nature of this study, there are inherent limitations. Second, artificial intelligent vessel extraction has limited capability for retinal vessel extraction in severe ROP with FEVR, which may result in some selection bias. In addition, compared to adults, fundus images of infants are more difficult to capture and have poorer quality, especially in terms of low contrast, uneven illumination and thinner vessels, thus the vessel segmentation was not performed as well as in adults. We look forward to future improvements in the algorithm and performance of artificial intelligence vascular extraction for more in-depth and comprehensive analysis.

In summary, the present study applied artificial intelligence for automated vessel extraction and compared the quantified morphological characteristics of the ROP, FEVR, and healthy full-term infants in fundus images. Differences were found between the three groups in vessel angle, vessel density, fractal dimension, and vascular tortuosity. Combined with artificial intelligence's vascular extraction capabilities, these vascular morphological characteristics, which were difficult to identify with the visual eye, would allow pediatric ophthalmologists to differentially diagnose the two diseases.

## Data Availability

The raw data supporting the conclusions of this article will be made available by the authors, without undue reservation.
